# Evaluation of microsatellite instability patterns in mismatch repair deficiency: a retrospective analysis of 285 endometrial cancers

**DOI:** 10.3389/fimmu.2025.1628979

**Published:** 2025-09-17

**Authors:** Cheng Wang, Min Feng, Yuling Kou, Wei Kuang, Wei Wang, Dongni Liang

**Affiliations:** ^1^ Department of Pathology, West China Second University Hospital, Sichuan University, Chengdu, Sichuan, China; ^2^ Key Laboratory of Birth Defects and Related Diseases of Women and Children, Ministry of Education, Chengdu, Sichuan, China

**Keywords:** minimal microsatellite shift, mismatch repair (MMR), microsatellite instability (MSI), endometrial cancer (EC), immunohistochemistry (IHC), polymerase chain reaction (PCR)

## Abstract

**Objective:**

In this study, we systematically compared the microsatellite shift patterns detected by PCR-based microsatellite instability analysis (PCR-MSI) in mismatch repair (MMR)-deficient ECs and analyzed the clinicopathological features associated with minimal versus major shifts.

**Method:**

We evaluated the microsatellite shift patterns using five NCI-recommended loci in 285 MMR-deficient ECs identified through immunohistochemistry (IHC). A minimal shift was defined as a one-to-three nucleotide repeat shift observed at least at one locus. Then, clinicopathological characteristics were analyzed for two distinct groups: the minimal shift group and the major shift group. Finally, further analysis of MMR/MSI discordant cases was performed through MLH1 promoter methylation detection and MMR gene germline mutation detection.

**Result:**

Of the 285 MMR-deficiency ECs, 169 (59.3%) had combined loss of MLH1 and PMS2, 54 (18.9%) had combined loss of MSH2 and MSH6, 39 (13.7%) had isolated loss of MSH6 and 23 (8.1%) had isolated loss of PMS2 by IHC. The rate of inconsistency between MMR-IHC and PCR-MSI was 12.3% (35/285). However, based on the minimal shifting criteria, 13 cases with MSI-L were reassessed as MSI-H because of the occurrence of minimal microsatellite shifts, and the inconsistency rate between MMR-IHC and MSI-PCR decreased to 7.7% (22/285). Additionally, discordant cases showed a higher frequency (91%, 20/22 cases) of minimal shift involving the mononucleotide locus. Among the 7 MLH1/PMS2-deficient cases, 3 were successfully detected and showed MLH1 promoter methylation. A total of 13 of 22 patients were successfully completed MMR gene germline testing, 11 cases had germline mutations in MSH6 and 3 cases harbored frameshift deletions (p.F1088Lfs*). Overall, the frequency of minimal shift was 100% (39/39) at isolated loss of MSH6, 85.8% (145/169) at the loss of MLH1 and PMS2, 66.7% (36/54) at the loss of MSH2 and MSH6, and 47.9% (11/23) at the isolated loss of PMS2, respectively. There is no correlation between minimal shift group or major shift group and clinicopathological features.

**Conclusion:**

MMR-deficient ECs exhibit a high frequency of minimal microsatellite shifts, particularly in cases with isolated loss of MSH6. The combination of MMR-IHC and MSI-PCR assays could enhance the accuracy of MSI detection, thereby facilitating more precise treatment strategies of ECs.

## Introduction

Microsatellite instability (MSI) refers to alterations in the length of short tandem repeats or microsatellites (MS) found throughout the genome. Insertion or deletion of repetitive units during DNA replication, as well as defects in the mismatch repair (MMR) system cause alterations in the length of microsatellite alleles, resulting in the associated molecular phenotype of MSI (1, 2). This replication error phenotype is thought to be a hallmark of hereditary cancer susceptibility syndromes that predispose patients to various types of cancer, particularly colorectal and endometrial cancer (EC) (3). Lynch syndrome (LS), also known as hereditary nonpolyposis colon cancer syndrome, is an autosomal-dominant inherited disorder caused by the pathogenic germline variant of MMR genes (including MLH1, PMS2, MSH2, MSH6 or EPCAM) (4). EC is the most prevalent extraintestinal tumor in women with Lynch syndrome (LS) and serves as a sentinel cancer for LS, with a lifetime risk of approximately 40-60% (5). MMR immunohistochemical (IHC) staining and MSI testing in EC were initially used for LS screening, which is identified in 3-5% of EC cases and is the most common hereditary cancer syndrome in EC (6). Regardless of the screening strategy, MLH1 promoter methylation triage is required to identify patients at highest risk for LS and explains the majority of defective MMR (dMMR) or MSI cases in EC (7).

Microsatellite instability-high (MSI-H) EC represents one of the four recognized molecular subtypes of EC and is associated with a relatively favorable prognosis (8). Consequently, testing for MMR or MSI is critical for molecular classification, prognostication of EC, screening for LS, and predicting responses to immunotherapy. Current guidelines from the College of American Pathologists (CAP) recommend that dMMR/MSI testing be conducted on all cases of ECs (9, 10). Although these assays exhibit a strong correlation, inconsistencies still exist in clinical practice (11). Compared to colorectal cancer (CRC), EC demonstrates a significantly higher frequency of minimal microsatellite shifts (1 to 3 nucleotide repeats at an affected locus) (12, 13). The objective of this study was to compare microsatellite shift patterns among deficiencies in MLH1, PMS2, MSH2, and MSH6 in ECs. Additionally, we aimed to analyze the correlation between the proportion of microsatellite shift patterns and clinicopathological characteristics, as well as the features of microsatellite patterns in discordant results.

## Materials and methods

### Specimens and patient data acquisition

**Figure 1:**
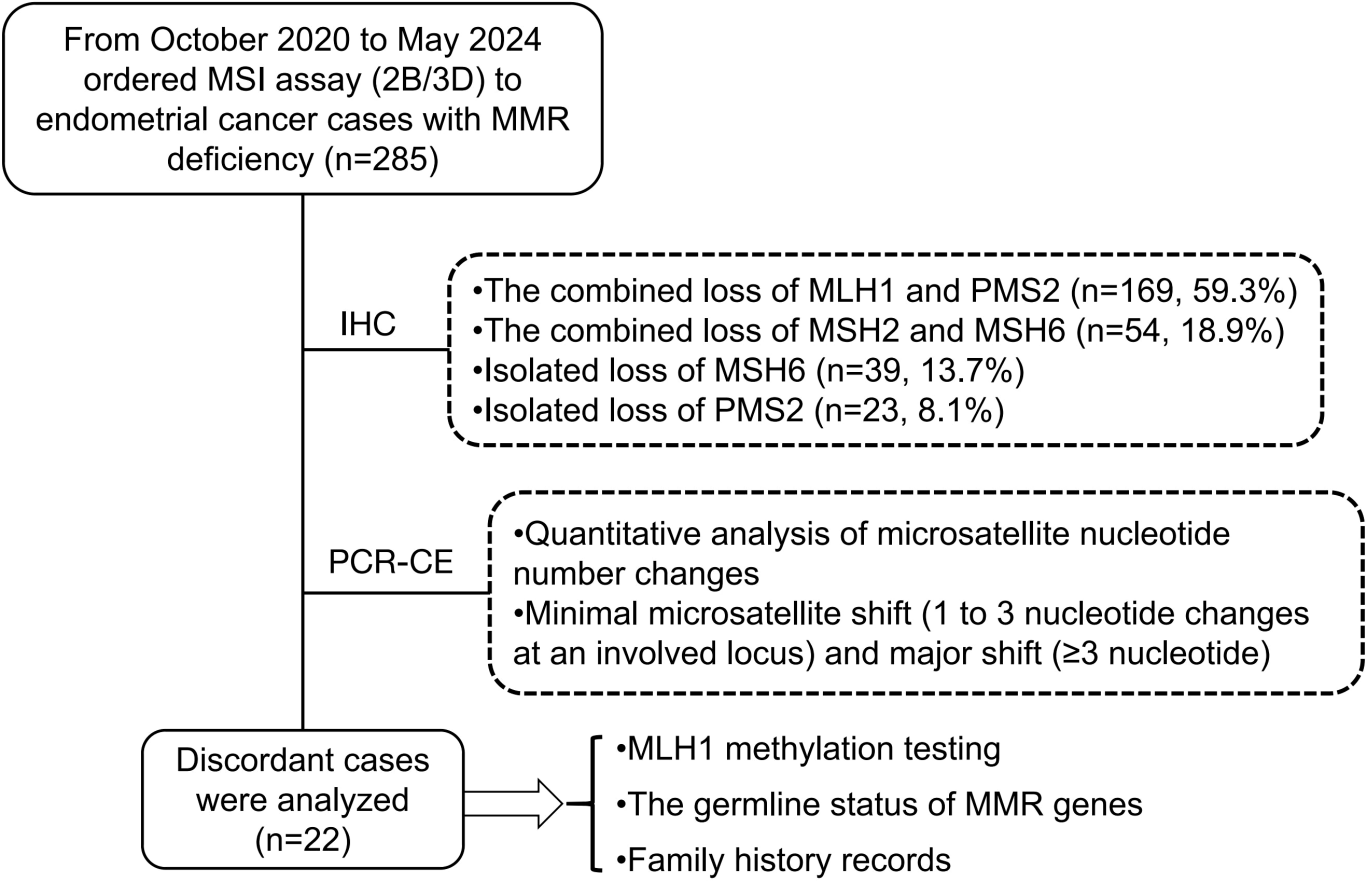
Flowchart of the study involving 285 eligible endometrial cancer.

Our retrospective study cohort included 285 endometrial cancer cases with mismatch repair protein deficiency, selected from the pathological system (PACS) at the Pathology Department of West China Second University Hospital. These cases consecutively underwent microsatellite instability PCR capillary electrophoresis testing between October 2020 and May 2024. Hematoxylin and eosin (H&E)-stained sections and mismatch repair protein immunohistochemical stains were reviewed by experienced gynecologic pathologists. Among the 285 dMMR ECs, the most common abnormal MMR-IHC result was the combined loss of MLH1 and PMS2 (169 cases, 59.3%), followed by the combined loss of MSH2 and MSH6 (54 cases, 18.9%), isolated loss of MSH6 (39 cases, 13.7%), and isolated loss of PMS2 (23 cases, 8.1%) (**Figure 1**). Clinicopathological features were extracted from electronic medical records and pathologic reports. PCR capillary electrophoresis (PCR-CE) data for microsatellite instability were reanalyzed for each case. This study was approved by the institutional review board of West China Second University Hospital [No. 2024 (181)]. All patient-identifying information was anonymized, and the study was conducted in accordance with the Declaration of Helsinki.

### MMR protein expression by immunohistochemistry

IHC staining was performed using BOND Polymer Refine Detection on Leica BOND-III (Leica Biosystems, Germany) according to the following major procedures:ER2 (PH8.4) antigen repair solution heat-induced epitope retrieval for 20 min; Peroxide Block incubation for 5 min, primary antibody incubation for 15 min, Post-Primary incubation for 8 min, Polymer incubation for 8 min, and mixed DAB incubation for 6 min; followed by hematoxylin counterstaining for 5 min. The primary antibodies, including MLH1 (1:100; clone ES05), PMS2 (1:100; clone EP51), MSH2 (1:1000; clone MX061), and MSH6 (1:1400; clone MX056), all sourced from Fuzhou Maixin Technology Co., Ltd., in Fuzhou, China. The staining outcomes were interpreted as follows: in the presence of clear internal (lymphocytes near tumor cells, fibroblasts cells or normal epithelial cells with nuclear staining) and external (appendix) positive controls, live tumor cells with clear nuclear staining were interpreted as having intact protein expression, whereas live tumor cells with clear absence of nuclear staining or focal weak nuclear staining were interpreted as having absent protein expression.

### MSI testing by PCR capillary electrophoresis

All endometrial carcinoma cases had PCR-MSI performed on the same tumor block analyzed by MMR-IHC. Hematoxylin & eosin staining slices were assessed for tumor cellularity and tumor cells (up to at least 30%) were enriched for DNA extraction. All tumor area and paired normal tissues were scraped using disposable surgical blade and put into 2mL-EP tube for digestion with protease K, UPure FFPE Tissue DNA Kit (Biokeyston, China) was used for DNA extraction according to instructions. MSI multiplex PCR assay contains fluorescently labeled primers like 2B/3D panel markers, BAT26/BAT25/D5S346/D17S250/D2S123/Penta C (Tongshu, China), the detail process as previous describe. Then, ABI 3500dx automated Genetic Analyzer was used for analyzing MSI status with GeneMapper IDX v.1.6 software. Tumor samples present nucleotide change in Allele peak (oriented left or right), compared to normal samples. MSI status was defined as MSI-high (MSI-H), which presented equal or more than to two instability loci; MSI-low (MSI-L) indicated only one locus, and microsatellite stability (MSS) showed no unstable loci.

### Quantitative analysis of microsatellite nucleotide number changes

**Figure 2:**
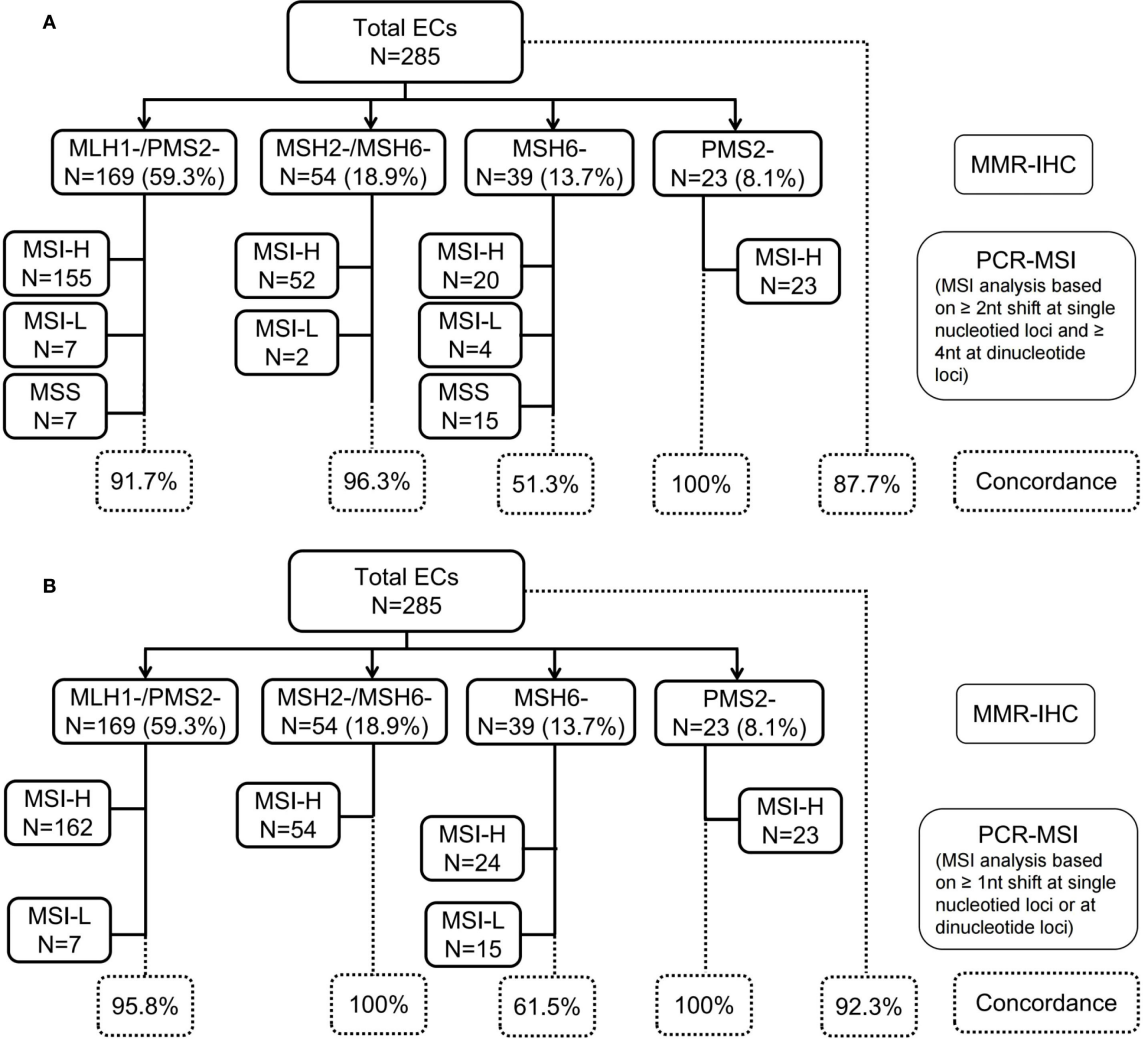
Profile of MMR-IHC and PCR-MSI (NCI-recommended loci) results from 285 cases of endometrial cancer. **(A)** MSI analysis based on ≥2 nucleotide (nt) shift at single nucleotide loci and ≥4 nt at dinucleotide loci; **(B)** MSI analysis based on ≥1 nt shift at single nucleotide loci or at dinucleotide loci. MMR, mismatch repair; IHC, immunohistochemistory; PCR, polymerase chain reaction; MSI, microsatellite instability; MSI-L, microsatellite instability-low; MSI-H, microsatellite instability-high; NCI, National Cancer Institute.

**Figure 3:**
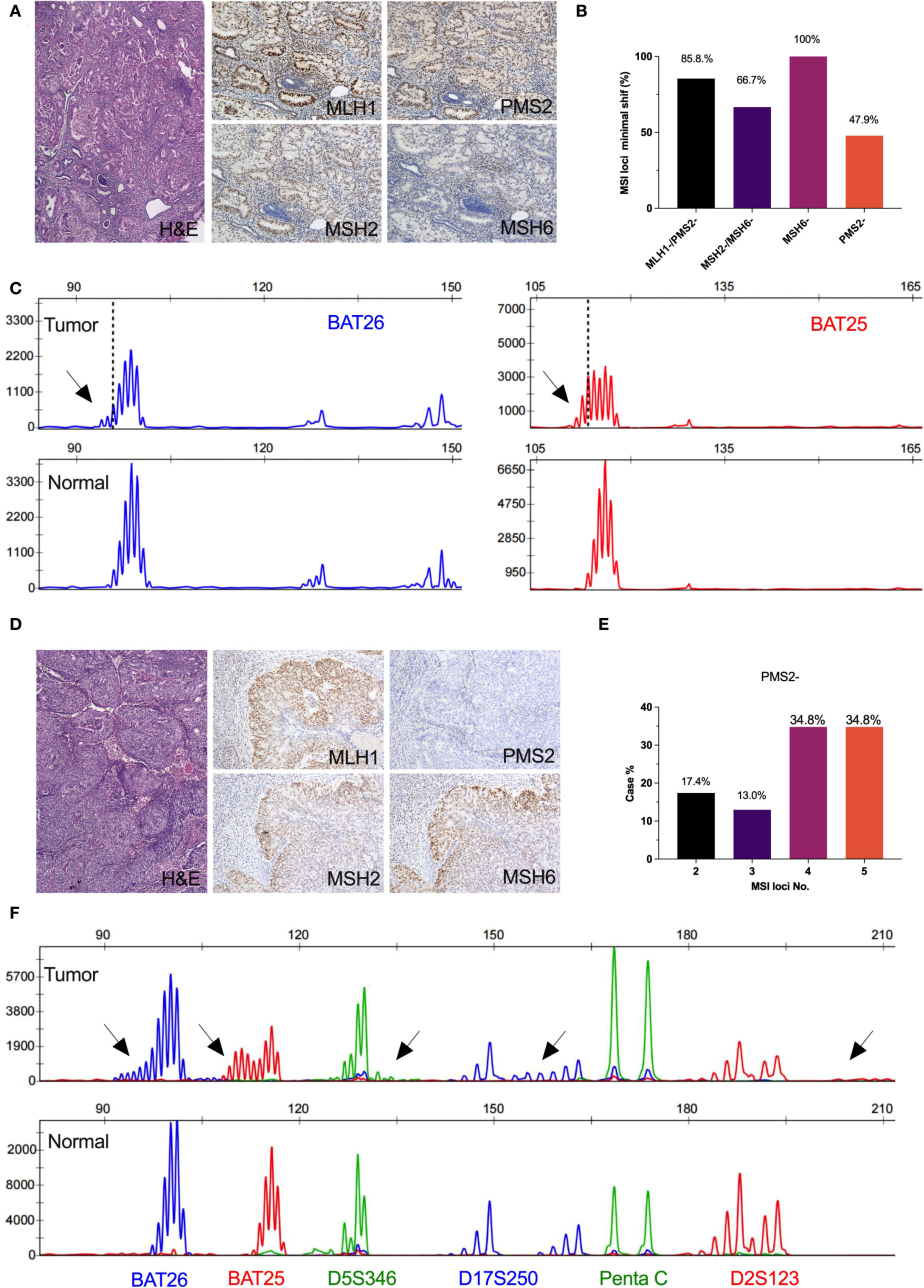
Microsatellite instability profiles in isolated loss of PMS2 or MSH6 immunohistochemical expression. **(A)** Isolated loss of MSH6 immunohistochemical expression; **(B)** The frequency of minimal microsatellite shifts in mismatch repair deficiency; **(C)** Minimal microsatellite shift at mononucleotide locus in a representative result of isolated loss of MSH6 case (BAT26, 2bp; BAT25, 2bp); **(D)** Isolated loss of PMS2 immunohistochemical expression; **(E)** The frequency of microsatellite instability number in isolated loss of PMS2; **(F)** Major microsatellite shift at mononucleotide loci (BAT26, 5bp; BAT25, 5bp)and dinucleotide loci (D5S346, 4bp; D17S250, 6bp; D2S123, 8bp) in a representative result of isolated loss of PMS2 case.

PCR capillary electrophoresis results were reviewed from 285 ECs by two molecular geneticists (C.W. and DN.L.). Compare the electropherogram of the tumor and normal samples from each patient, marking the leftmost or rightmost valid peak (valid peak height: <5% of the highest peak) in 2B/3D marker loci. Record the difference in the size of the leftmost or rightmost peak between the tumor and paired samples, which is defined as an absolute nucleotide shift representing the length of the nucleotide repeat of the tumor sample. According to the kit definition (**Figure 2A**), single nucleotide loci were judged to be unstable when it showed ≥2 nucleotide or base changes (≥4 for dinucleotide loci). When compared between the tumor and its paired normal tissue, MSI-H was defined by the presence of microsatellite instability at ≥2 loci, MSI-L indicated only one locus, and MSS showed no unstable loci. In addition, we further reviewed the minimal microsatellite shift (**Figure 2B**), and reinterpreted nucleotide loci as unstable when they showed ≥1 nucleotide or base change, either mono- or dinucleotide. Minimal microsatellite shift (**Figure 3C**) referred to 1 to 3 nucleotide changes at an involved locus, and major shift (**Figure 3F**) was defined by ≥3 nucleotide or base change.

### Analysis of MMR/MSI discordant EC cases through MLH1 promoter methylation detection and MMR gene germline mutation detection

Next Generation Sequencing (NGS) was performed using the Geneplus platform: library construction, target capture, sequencing and bioinformatics analysis were performed as previous described (14). The multi-gene NGS panel was utilized, including MLH1, MSH2, MSH6, PMS2, EPCAM, etc. In brief, NGS panel detected single nucleotide variants (SNVs), insertions and deletions (InDels), and copy number alterations (CNVs) of the assessed genes. Additionally, tumors with MLH1-deficient cases on IHC underwent MLH1 promoter methylation testing using methylation-specific PCR. Genomic DNA was extracted from FFPE tumor tissue specimens (with at least 30% cellularity), followed by bisulfite treatment of tumor DNA for amplification via fluorescence real-time PCR detection, with a detailed procedure described in our previous studies (11).

### Statistical analysis

The data were analyzed with Graphpad Prism version 9.0 software. We used the Chi-square test or Fisher’s exact test to compare the distribution of clinicopathological variables between proportion of minimal shift set (minimal shift No./instable loci No. >50%) and major shift set (minimal shift No./instable loci No. ≤50%). The data were shown as frequency descriptions and ratios. In the present study, *P* value <0.05 was identified as statistically significant.

## Results

### Clinicopathological features of ECs with MMR deficiency

A total of 1,088 patients were diagnosed with EC at West China Second University Hospital between October 2020 and May 2024. Among them, 285 patients (26.2%) with dMMR were included in this study. The study cohort consisted of 249 cases (87.4%) of endometrioid carcinoma, 19 cases (6.7%) of mixed carcinoma, 7 cases (2.5%) of undifferentiated and dedifferentiated endometrial carcinoma, 6 cases (2.1%) of carcinosarcoma, and 4 cases (1.4%) of clear cell carcinoma. The median age of the patients was 54 years (range: 32–82 years). A total of 83.5% of the patients were diagnosed at an early stage (FIGO I–II). There were 100 (35.1%) patients with lymph-vascular space invasion, and 34 patients (11.9%) developed lymphatic metastases. Of the 285 patients with dMMR, 169 (59.3%) showed loss of expression of MLH1 and PMS2, and 53 (18.9%) showed loss of expression of MSH2 and MSH6, and 39 (13.7%) and 23 (8.1%) patients showed the solely loss of expression of MSH6 and PMS2, respectively. Detailed patient characteristics are presented in **Table 1**.

**Table 1:** The correlation of clinicopathological features with the proportion of minimal shift set (minimal shift No./instable loci No. >50%) and large shift set (minimal shift No./instable loci No. ≤50%) in endometrial cancers.

Clinicopathological parameter	Total	Minimal shift set	Large shift set	*P*
	(n = 285)	(n = 102)	(n = 183)	
Age				0.122
Median [Min, Max]	54.0 [32.0, 82.0]	54.4 [35.0, 71.0]	53.7 [32.0, 82.0]	
<55	164 (57.5%)	52 (51.0%)	112 (61.2%)	
≥55	121 (42.5%)	50 (49.0%)	71 (38.8%)	
Histology				0.193
Endometrioid carcinoma	249 (87.4%)	91 (89.2%)	158 (86.3%)	
Mixed carcinoma	19 (6.7%)	9 (8.8%)	10 (5.5%)	
Undifferentiated and dedifferentiated carcinoma	7 (2.5%)	1 (1.0%)	6 (3.3%)	
Carcinosarcoma	6 (2.1%)	0 (0%)	6 (3.3%)	
Clear cell carcinoma	4 (1.4%)	1 (1.0%)	3 (1.6%)	
Grade				0.980
G1/2	214 (75.1%)	76 (74.5%)	138 (75.4%)	
G3	71 (24.9%)	26 (25.5%)	45 (24.6%)	
FIGO				0.576
I-II	238 (83.5%)	83 (81.4%)	155 (84.7%)	
III-IV	47 (16.5%)	19 (18.6%)	28 (15.3%)	
LVSI				1.000
No/Unknown	185 (64.9%)	66 (64.7%)	119 (65.0%)	
Yes	100 (35.1%)	36 (35.3%)	64 (35.0%)	
Myometrial invasion				1.000
Superficial	226 (79.3%)	81 (79.4%)	145 (79.2%)	
Deep	29 (20.7%)	21 (20.6%)	38 (20.8%)	
Lymphatic metastasis				0.799
No/Unknown	251 (88.1%)	91 (89.2%)	160 (87.4%)	
Yes	34 (11.9%)	11 (10.8%)	23 (12.6%)	
MMR protein				<0.001
MLH1-/PMS2-	169 (59.3%)	60 (58.8%)	109 (59.6%)	
MSH2-/MSH6-	53 (18.9%)	11 (10.8%)	43 (23.5%)	
MSH6-	39 (13.7%)	30 (29.4%)	9 (4.9%)	
PMS2-	23 (8.1%)	1 (1.0%)	22 (12.0%)	
Consistency of MMR/MSI				<0.001
Discordant (dMMR/MSI-L)	22 (7.7%)	20 (19.6%)	2 (1.1%)	
Concordant (dMMR/MSI-H)	263 (92.3%)	82 (80.4%)	181 (98.9%)	

LVSI, Lymph-vascular space invasion; MMR, Mismatch repair; dMMR, deficient MMR; MSI-L, Microsatellite instability low; MSI-H, Microsatellite instability high.

### Microsatellite instability patterns associated with MMR-deficiency in ECs

PCR-MSI analysis, based on single nucleotide loci with shifts of ≥2 nucleotides and dinucleotide loci with shifts of ≥4 nucleotides, classified 87.7% (250/285) of cases as MSI-H, 4.6% (13/285) MSI-L, and 7.7% (22/285) MSS. The discordance rate between MMR-IHC and PCR-MSI was 12.3% (35/285) (**Figure 2A**). However, upon reevaluation using the minimal shift criterion, 13 MSI-L cases were reclassified as MSI-H due to the presence of minimal microsatellite shifts, thereby increasing the concordance rate between MMR-IHC and MSI-PCR from 87.7% to 92.3% (**Figure 2B**). Notably, the highest discordance rate between MMR-IHC and MSI-PCR was observed in cases with isolated loss of MSH6, remaining at 38.5% even after incorporating the minimal shift interpretation criteria.

**Figure 4:**
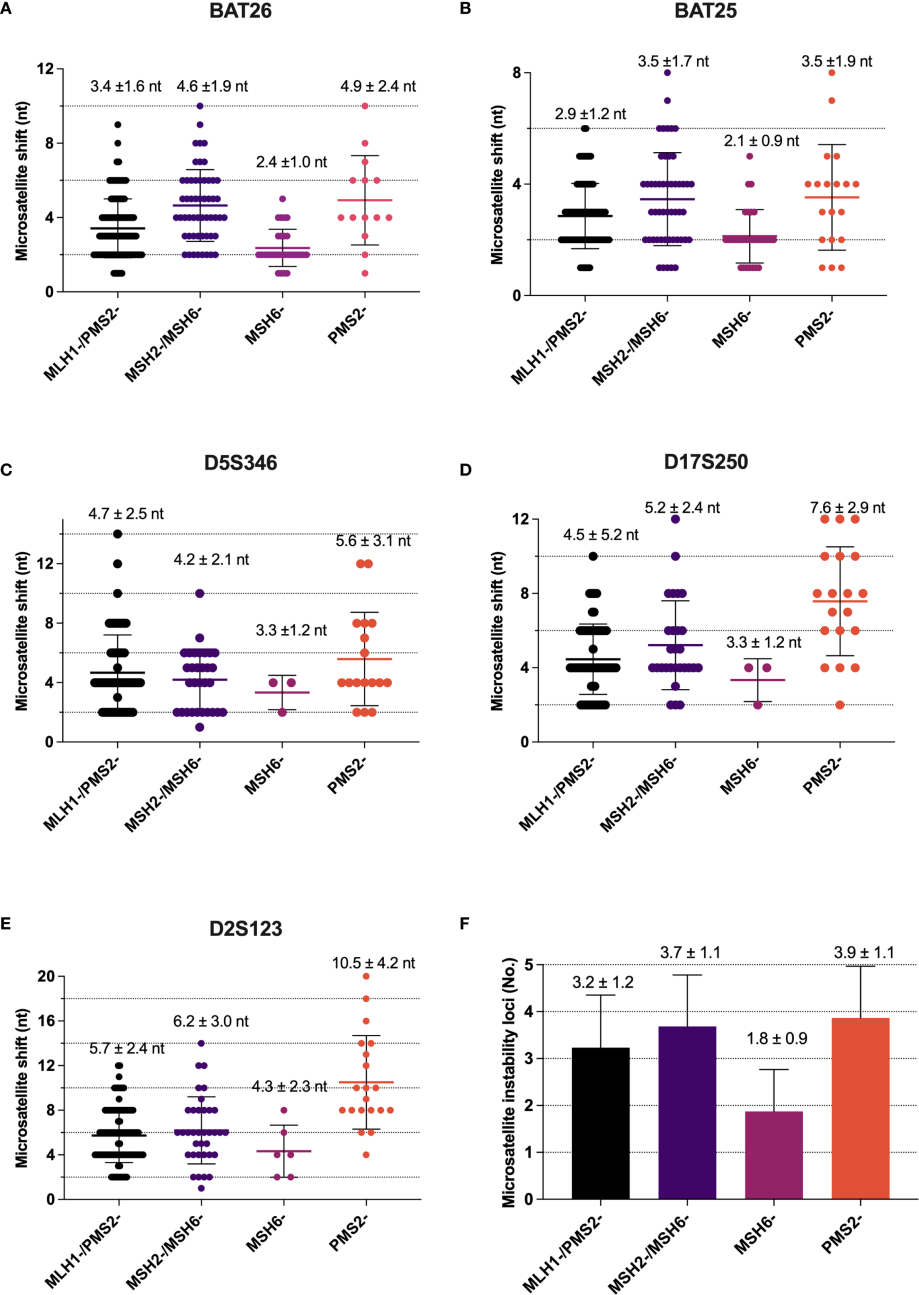
Difference in microsatellite shift between MLH1, PMS2, MSH2 and MSH6 deficiency. **(A)** Alterations in repeat length in BAT26 locus; **(B)** Alterations in repeat length in BAT25 locus; **(C)** Alterations in repeat length in D5S346 locus; **(D)** Alterations in repeat length in D17S250 locus; **(E)** Alterations in repeat length in D2S123 locus; **(F)** The number of microsatellite instability loci in MMR protein deficiency (mean ± SD).

PCR-MSI results revealed a significant reduction in the mean number of the microsatellite repeats across the five loci in the group with isolated MSH6 loss compared to the group with isolated PMS2 loss (p<0.001, **Figures 4A–E**). Among the combined loss groups, all exhibited more than three microsatellite repeats, except for the MLH1/PMS2 combined loss group, which demonstrated an average repeat count of fewer than three nucleotides at the BAT25 locus. Moreover, the mean number of microsatellite instability loci was significantly lower in the isolated MSH6 loss group (mean ± SD, 1.8 ± 0.9) compared to other groups (p<0.001, **Figure 4F**). Additionally, single nucleotide loci were more prone to minimal shifts compared to dinucleotide loci, with the following frequencies observed: BAT26 50.1%, BAT25 58.5%, D5S346 12.2%, D17S250 8.4%, and D2S23 7.0% (**Supplementary Table 1**).

Overall, 81.1% (231/285) of MSI cases associated with dMMR in ECs exhibited minimal microsatellite shifts. Specifically, the frequency of minimal shifts was observed as follows: 62.8% (145/231) in cases with combined loss of MLH1 and PMS2, 16.9% (39/231) in cases with isolated loss of MSH6, 15.6% (36/231) in cases with combined loss of MSH2 and MSH6, and 4.7% (11/231) in cases with isolated loss of PMS2 (Supplementary **Table 2**). Interestingly, all cases (100%, 39/39) of EC patients with MSH6-deficiency demonstrated minimal shifts, while less than half (47.9%, 11/23) of cases with PMS2-deficiency exhibited this characteristic (**Figure 3B**). Representative microsatellite profiles for these patterns are shown in **Figures 3C, F**, where cases with isolated loss of MSH6 (**Figure 3A**) or PMS2 (**Figure 3D**) present distinctly different shift patterns. Additionally, nearly 70% of cases with isolated loss of PMS2 exhibited instability at more than three loci (**Figure 3E**).

**Table 2:** Different microsatellite shifts in endometrial caners of discordant cases (N = 22).

Case code	Age	Family history	MSI loci shift	Minimal shift (%)	MMR protein deficiency	MLH1 methylation	Germline variations
1	58	No/Unknown	BAT26, 1bp	100%	MLH1-/PMS2-	Yes	–
2	57	No/Unknown	BAT25, 1bp	100%	MSH6-	–	MSH6, p.S602Kfs*
3	58	No/Unknown	BAT25, 3bp	100%	MSH6-	–	MSH6, p.Q1048*
4	48	MU, CRC; ES, EC; PU, LIC	BAT26, 1bp	100%	MSH6-	–	MSH6, p.K218Kfs*
5	67	No/Unknown	BAT25, 2bp	100%	MSH6-	–	MSH6, p.F1088Lfs*
6	66	No/Unknown	BAT26, 2bp	100%	MLH1-/PMS2-	NA	–
7	51	No/Unknown	BAT26, 2bp	100%	MLH1-/PMS2-	NA	–
8	57	No/Unknown	BAT25, 2bp	100%	MSH6-	–	None
9	53	M, BC	BAT25, 1bp	100%	MLH1-/PMS2-	Yes	–
10	53	No/Unknown	BAT26, 2bp	100%	MSH6-	–	MSH6, p.K125*
11	45	No/Unknown	D2S123, 4bp	0	MLH1-/PMS2-	NA	–
12	49	No/Unknown	BAT26, 1bp	100%	MSH6-	–	–
13	55	F, LUC	BAT25, 2bp	100%	MSH6-	–	MSH6, R240*
14	46	No/Unknown	D2S123, 6bp	0	MLH1-/PMS2-	Yes	–
15	48	ES, EC	BAT25, 1bp	100%	MSH6-	–	MSH6, pE288*
16	56	ES, EC	BAT26, 1bp	100%	MSH6-	–	MSH6, c.3646 + 1del
17	48	No/Unknown	BAT25, 1bp	100%	MSH6-	–	MSH6, p.Q132Vfs*
18	56	No/Unknown	BAT25, 2bp	100%	MSH6-	–	MSH6, p.F1088Lfs*
19	46	F, CRC and LYP	BAT26, 2bp	100%	MSH6-	–	–
20	59	F, LIC	BAT25, 1bp	100%	MSH6-	–	None
21	57	No/Unknown	BAT26, 2bp	100%	MSH6-	–	MSH6, p.F1088Lfs*
22	58	M, GC	BAT25, 1bp	100%	MLH1-/PMS2-	NA	–

MMR, Mismatch repair; *Loci minimal shift %, minimal shift No./instable loci No.; M, mother; YB, younger brother; ES, elder sister; MU, maternal uncle; PU, paternal uncle; LIC, liver cancer; CRC, colorectal cancer; EC, endometrial cancer; LUC, lung cancer; BC, breast carcinoma; LYP, lymphoma; GC, gastric cancer.; NA, not available.

### Correlation between clinicopathological features and minimal microsatellite instability

In this study, 81.1% (231 of 285 cases) of MMR-deficient ECs exhibited minimal microsatellite shifts. We further compared the clinicopathological characteristics between the minimal shift group (minimal shift/major shift >50%) and the large shift group (minimal shift/major shift ≤50%). No significant differences were observed in age, histology, grade, FIGO stage, lymphovascular invasion, or lymph node metastasis (p >0.05; **Table 1**). However, the expression patterns of MMR proteins differed significantly between the two groups. Specifically, the large shift group demonstrated a higher proportion of isolated PMS2 loss (12% vs. 1%, p <0.001) and a lower proportion of isolated MSH6 loss (4.9% vs. 29.4%, p <0.001) compared to the minimal shift group. As expected, the large shift group showed higher concordance with MMR deficiency (80.4% vs. 98.9% in the minimal shift >50% and minimal shift ≤50% groups, respectively; p <0.001). Conversely, the minimal shift group exhibited a significantly higher rate of discordant cases compared to the large shift group (19.9% vs. 1.1%, p <0.001).

### The characteristics of microsatellite patterns in discordant cases

In discordant cases, 8 of the 22 (36.4%) patients had a family history, including CRC, EC, liver cancer, lung cancer, breast carcinoma, lymphoma and gastric cancer. Among these, three-quarters of cases (75%) were presented the isolated loss of MSH6, while the combined loss of MLH1 and PMS2 accounted for 25% (2 out of 8 cases) (**Table 2**). MLH1 promoter methylation testing was performed on MLH1-deficient cases. Among the 7 cases, 3 were successfully detected and showed MLH1 promoter methylation. A total of 13 of 22 patients were successfully completed further MMR gene germline testing. Among the 13 patients, 11 cases had germline mutations in MSH6, three cases exhibited the same mutation site (MSH6, p.F1088Lfs*). Although both minimal and major microsatellite shifts were observed in a certain proportion of cases with abnormal MMR proteins, discordant cases exhibited a significantly higher frequency (91%, 20 out of 22 cases) of minimal shifts involving mononucleotide loci. Notably, only two cases demonstrated combined loss of MLH1 and PMS2 with large minimal shifts (>3 nucleotides) (**Table 2**).

## Discussion

Here, we evaluated the minimal microsatellite shift in the NCI-recommended microsatellite instability assay for its application in endometrial cancer using PCR-capillary electrophoresis. This evaluation was based on a retrospective analysis of 285 MMR-deficient ECs. Our findings indicate that MMR-deficient ECs exhibit a high frequency of minimal microsatellite shifts. Additionally, distinct patterns of microsatellite shifts were observed among the four MMR protein expressions, with notably different results for isolated losses of MSH6 compared to PMS2.

Detecting MSI status is critical for the management of cancer patients, as it serves as a predictor for Lynch syndrome irrespective of the primary tumor type and also predicts the response to immune checkpoint inhibitors. This is particularly relevant in identifying molecular subtypes of endometrial cancer (2). While current literature has explored the concordance between MMR-IHC and PCR-MSI, many studies suffer from limitations such as small sample sizes and a lack of large population-based analyses for specific cancers (15, 16). A study analyzing 50 MSI-H cases of endometrial carcinoma identified microsatellite shift patterns, revealing that 52% of MSI-H cases exhibited minimal microsatellite shifts, defined as one-to-three nucleotide repeat shifts at the involved loci (13). As the largest gynecological oncology center in Southwest China, we have collected 285 endometrial cancer cases with dMMR and reanalyzed their microsatellite shift patterns using a standardized approach conducted by two molecular pathologists. Our findings confirm that minimal shifts are a prevalent phenomenon, with their frequency correlating with the expression patterns of MMR proteins. In our study, 81.1% of MMR-deficient ECs exhibited minimal microsatellite shifts. Specifically, the frequency of minimal shifts was 62.8% in cases with combined loss of MLH1 and PMS2, 16.9% in cases with isolated loss of MSH6, 15.6% in cases with combined loss of MSH2 and MSH6, and 4.7% in cases with isolated loss of PMS2. These findings are consistent with those reported by Wu et al., who also observed the highest frequency of combined loss of PMS2 and MLH1 (13). However, there were significant variations in the proportions of minimal microsatellite shifts among the four MMR-deficient patterns. Notably, all cases (100%) with isolated loss of MSH6 demonstrated minimal shifts, whereas only 47.9% of cases with isolated loss of PMS2 exhibited this characteristic. Therefore, these results indicate that neglecting minimal shifts may lead to misinterpretation of microsatellite instability in clinical diagnostics.

In prior comparative analyses of MSI in endometrial and colorectal cancers, it was observed that the size of deletions/insertions differed between these two cancer types. Specifically, ECs exhibited microsatellites with shorter alterations or smaller nucleotide shifts compared to CRCs (17–19). Our findings further revealed that MSI-H ECs frequently demonstrated microsatellite sequences with minor shifts. Additionally, isolated loss of MSH6 was associated with a minimal shift pattern, which could potentially be misclassified as MSI-L or MSS tumors. These observations underscore distinct pathogenic mechanisms underlying MSI-H endometrial and colorectal cancers and highlight the critical importance of detecting minimal microsatellite shifts for accurate interpretation. According to the recommendations of the College of American Pathologists (CAP), immunohistochemistry for MMR protein testing is preferred over PCR-MSI or NGS methods for patients with EC being considered for immune checkpoint inhibitor therapy (20). This recommendation is based on the widespread availability of immunohistochemical analysis in most laboratories, its lower cost, and high concordance with molecular testing results. Similarly, the ASCO’s diagnostic guidelines for immunotherapy recommend MMR-IHC, MSI-PCR, and NGS testing for colorectal cancer; MMR-IHC and MSI-PCR testing for other gastrointestinal cancers (including colorectal, gastric, and esophageal cancers); and solely MMR-IHC testing for ECs (3, 15). Considering the differences in sample types (such as curettage or hysterectomy specimens), specimen pre-processing conditions (such as ischemia duration or fixation time), and IHC staining techniques (such as antigen retrieval methods or clone selection), MMR-deficient ECs typically exhibit different combinations of proportion, intensity, and MMR protein expression patterns. Particularly for EC patients with subclonal loss of MMR protein expression, there are still practical challenges in minimizing misjudgment or indeterminate results (21). Therefore, the combined application of MMR-IHC and MSI-PCR is the most sensitive and specific method for identifying dMMR tumors.

Previous studies have indicated ECs exhibiting minimal microsatellite shifts may involve complex underlying mechanisms, including differences in tumor cell biology, the MMR system, and varying stages of tumor progression. Early-stage tumors may undergo less DNA replication and consequently exhibit fewer microsatellite shifts compared to advanced-stage tumors (22). In cases of isolated MSH6 loss, the MSH3 protein can partially compensate for the repair function of MSH6, and the MSH2/MSH3 heterodimer retains a DNA repair capacity that prevents significant DNA accumulation, resulting in a non-MSI-H status. Furthermore, minimal microsatellite shifts were more likely to occur when the proportion of tumor cells in tested samples was less than 30%. PCR-CE using dinucleotide markers (D2S123, D17S250, D5S346) tended to produce more minimal shifts compared to single nucleotide markers (BAT-25, BAT-26, MONO-27) (21). In scenarios with low tumor cell ratios, laser microdissection can be employed to enhance the accuracy of analysis.

There is indeed inconsistency between MMR-IHC and PCR-CE MSI testing, with 1–3% of cases reported in the CRC literature and slightly higher proportions of 3–7% reported in the EC literature (7). MSI analysis has been shown to be more reliable in CRCs than in ECs due to the predominant occurrence of major microsatellite shifts in CRCs. Moreover, the MSI peak shifts depend on the specific MMR gene involved, with hereditary MSH6-deficient CRCs having shorter MSI peak lengths than other MMR-deficient CRCs (23). A previous study demonstrated a high concordance rate of 98.8% between MMR-IHC staining and MSI analysis; however, discordant cases in ECs predominantly exhibited MSH6 deficiency (11). Importantly, non-MSI-H pattern and loss of MSH6 is the most frequently observed discordancy (24). Tumors carrying *MSH6* germline mutations are prone to exhibit inconsistent MSI and/or IHC phenotypes compared with other MMR genes, primarily due to partial redundancy of the function of MSH6 and MSH3. Wang et al. (14) found that carriers of germline pathogenic MSH6 variants are more likely to develop EC at an older age, and that the non-MSI-H phenotype with minimal microsatellite shifts was frequently observed only when MSH6 protein loss. Therefore, this atypical MSI pattern is often overlooked, potentially increasing the risk of LS misdiagnosis. In our study, among the discordant cases, isolated MSH6 loss accounted for 68.2% (15 of 22 cases), with an average absolute shift of one to two nucleotide repeats (minimal shift). Thirteen patients successfully completed further MMR gene germline mutation testing, with 11 cases showing germline mutations in the MSH6 gene, including 3 cases showing the same mutation site (MSH6, p.F1088Lfs*). Truncation mutations can introduce premature stop codons, resulting in a truncated C-terminal form of the protein. The complete or partial loss of this domain leads to the loss of ATPase activity, thereby impairing DNA binding and mismatch repair functions (3). Even so, PCR-MSI detection methods cannot truly identify the MSI pattern of MSH6 germline mutations, especially at the 2B/3D panel. So, the five loci recommended by the NCI may not provide the most sensitive amplicons for PCR-based MSI assays. Recent studies have advocated that the Pentaplex panel or Promega panel could enhance detection sensitivity in ECs using PCR-MSI assay (21, 25). Currently, assessing MSI pattern in EC may be more challenging because microsatellite shifts are more subtle. Therefore, Bethesda (2B/3D panel) and Promega (mononucleotide panel) testing results required professionally trained molecular geneticists to overcome interpretation complexities caused by minimal shifts in microsatellite repeat length.

Collectively, these findings indicate that MMR-IHC and PCR-MSI based techniques are not as equivalent as previously assumed. Even a relatively high concordance rate might not suffice for routine clinical diagnostics. Factors contributing to these discrepancies include technical and pre-analytical variables such as tissue ischemia-hypoxia time and inadequate fixation. Notably, current research highlights that tumors in MSH6 variant carriers exhibit a higher propensity for discordant MSI and/or MMR phenotypes compared to those associated with other MMR genes (26, 27). Pathologists and clinicians should be aware that germline MSH6 variant carriers often present with minimal shift patterns and recognize that discordant cases (isolated loss of MSH6 without MSI-H) represent an essential step in the genetic diagnosis of Lynch syndrome. Additionally, the MSS/MSI-L with MMR-deficient phenotype, potentially caused by hypermethylation of the MLH1 promoter or somatic MMR gene mutations (11), warrants attention. Interestingly, we performed MLH1 promoter methylation testing on discordant MSI and/or IHC phenotypes with MLH1 deficiency and successfully detected MLH1 promoter methylation in three cases. Conversely, isolated loss of PMS2 expression is considered a rare phenotype in colorectal cancers, accounting for approximately 4% of MSI-positive tumors in Western populations and 7.9% in southern China (28, 29). Further investigation into the clinical characteristics and underlying causes of this high proportion of isolated PMS2 loss would be valuable. Importantly, isolated loss of PMS2 staining is not exclusively indicative of germline PMS2 mutations; some patients with this phenotype harbor germline mutations or promoter hypermethylation in MLH1 rather than PMS2, as germline point mutation.

Remarkably, our findings indicate that isolated PMS2 loss is predominantly associated with large microsatellite shifts, where over 80% of unstable loci exhibit shifts greater than three, encompassing both mononucleotide and dinucleotide repeats. However, whether this microsatellite shift pattern can serve as a reliable indicator for isolated PMS2 loss or PMS2-associated Lynch syndrome requires further investigation. Minimal microsatellite shifts may be easily overlooked or misdiagnosed as false-negative microsatellite stability results using PCR capillary electrophoresis in EC patients. This issue can be effectively addressed through the integration of MMR-IHC and PCR-MSI testing strategies. Furthermore, in EC cases with MSI-H, no significant differences were observed in clinicopathological features between minimal and large shift patterns. Nevertheless, the shifting patterns could potentially reflect the subtype specificity of MMR-deficient, such as distinguishing MSH6 from PMS2. The correlation between microsatellite shift patterns and the tumor microenvironment or treatment response warrants further exploration in future studies by leveraging multi-omics data. In particular, the clinical and pathological characteristics of patients with microsatellite minimal shift tumors merit in-depth investigation, including whether their immune microenvironment is similar to that of major shift tumors, as well as differences in immunotherapy and prognosis. Additionally, to fully realize clinical potential, automated tools must be developed to calculate MSI peak shift lengths from PCR-based fragment length data, thereby eliminating subjective influences caused by variations in manual counting.

## Conclusion

MMR-deficient ECs exhibit a higher frequency of minimal microsatellite shifts in cases of isolated loss of MSH6, which differs from the large microsatellite shifts associated with isolated loss of PMS2. Additionally, MMR-deficient ECs show a higher incidence of isolated loss of MSH6. Diagnostically, PCR-based MSI assays demonstrate reduced sensitivity in cases of isolated MSH6 loss, and minimal microsatellite shifts may represent a potential diagnostic pitfall in assessing microsatellite instability in ECs. Minimal shifts are also the primary cause of inconsistent results between MMR-IHC and MSI-PCR assays. Therefore, interpretation should account for minor microsatellite shifts, and complementary combined detection using both MMR-IHC and MSI-PCR represents the most sensitive and specific approach for identifying MMR-deficient tumors.

## Data Availability

The original contributions presented in the study are included in the article/**Supplementary Material**. Further inquiries can be directed to the corresponding author.

## References

[1] Chambers GK MacAvoy ES. Microsatellites: consensus and controversy. Comp Biochem Physiol B Biochem Mol Biol. (2000) 126:455–76. doi: 10.1016/s0305-0491(00)00233-9, PMID: 11026658

[2] Latham A Srinivasan P Kemel Y Shia J Bandlamudi C Mandelker D. Microsatellite instability is associated with the presence of lynch syndrome pan-cancer. J Clin Oncol. (2019) 37:286–95. doi: 10.1200/JCO.18.00283, PMID: 30376427 PMC6553803

[3] van der Werf-’t Lam A-S Terlouw D Tops CM van Kan MS van Hest LP Gille HJP. Discordant staining patterns and microsatellite results in tumors of MSH6 pathogenic variant carriers. Mod Pathol. (2023) 36:100240. doi: 10.1016/j.modpat.2023.100240, PMID: 37307877

[4] Peltomäki P Nyström M Mecklin J-P Seppälä TT. Lynch syndrome genetics and clinical implications. Gastroenterology. (2023) 164:783–99. doi: 10.1053/j.gastro.2022.08.058, PMID: 36706841

[5] Aarnio M Sankila R Pukkala E Salovaara R Aaltonen LA de la Chapelle A. Cancer risk in mutation carriers of DNA-mismatch-repair genes. Int J Cancer. (1999) 81:214–8. doi: 10.1002/(sici)1097-0215(19990412)81:2<214::aid-ijc8>3.0.co;2-l 10188721

[6] Wang Y Wang Y Li J Cragun J Hatch K Chambers SK. Lynch syndrome related endometrial cancer: clinical significance beyond the endometrium. J Hematol Oncol. (2013) 6:22. doi: 10.1186/1756-8722-6-22, PMID: 23531335 PMC3623651

[7] Riedinger CJ Esnakula A Haight PJ Suarez AA Chen W Gillespie J. Characterization of mismatch-repair/microsatellite instability-discordant endometrial cancers. Cancer. (2024) 130:385–99. doi: 10.1002/cncr.35030, PMID: 37751191 PMC10843110

[8] Cancer Genome Atlas Research Network Kandoth C Schultz N Cherniack AD Akbani R Liu Y. Integrated genomic characterization of endometrial carcinoma. Nature. (2013) 497:67–73. doi: 10.1038/nature12113, PMID: 23636398 PMC3704730

[9] Liu YL Weigelt B. A tale of two pathways: Review of immune checkpoint inhibitors in DNA mismatch repair-deficient and microsatellite instability-high endometrial cancers. Cancer. (2024) 130:1733–46. doi: 10.1002/cncr.35267, PMID: 38422006 PMC11058027

[10] Koh W-J Abu-Rustum NR Bean S Bradley K Campos SM Cho KR. Uterine neoplasms, version 1.2018, NCCN clinical practice guidelines in oncology. J Natl Compr Canc Netw. (2018) 16:170–99. doi: 10.6004/jnccn.2018.0006, PMID: 29439178

[11] Wang C Kuang W Zeng J Ren Y Liu Q Sun H. A retrospective study of consistency between immunohistochemistry and polymerase chain reaction of microsatellite instability in endometrial cancer. PeerJ. (2023) 11:e15920. doi: 10.7717/peerj.15920, PMID: 37663290 PMC10470453

[12] Wang Y Shi C Eisenberg R Vnencak-Jones CL. Differences in microsatellite instability profiles between endometrioid and colorectal cancers: A potential cause for false-negative results? J Mol Diagn. (2017) 19:57–64. doi: 10.1016/j.jmoldx.2016.07.008, PMID: 27810331 PMC5225298

[13] Wu X Snir O Rottmann D Wong S Buza N Hui P. Minimal microsatellite shift in microsatellite instability high endometrial cancer: a significant pitfall in diagnostic interpretation. Mod Pathol. (2019) 32:650–8. doi: 10.1038/s41379-018-0179-3, PMID: 30443012

[14] Wang C Liang D Wang W Kuang W Zou J Zeng J. Early-stage endometrioid carcinoma with MSH6 protein deficiency: pitfalls in the diagnostic interpretation of microsatellite instability. Front Oncol. (2025) 15:1520500. doi: 10.3389/fonc.2025.1520500, PMID: 40469174 PMC12133529

[15] Nádorvári ML Lotz G Kulka J Kiss A Tímár J. Microsatellite instability and mismatch repair protein deficiency: equal predictive markers? Pathol Oncol Res. (2024) 30:1611719. doi: 10.3389/pore.2024.1611719, PMID: 38655493 PMC11036414

[16] Wang C Zhang L Vakiani E Shia J. Detecting mismatch repair deficiency in solid neoplasms: immunohistochemistry, microsatellite instability, or both? Mod Pathol. (2022) 35:1515–28. doi: 10.1038/s41379-022-01109-4, PMID: 35668150

[17] Chung Y Nam SK Chang HE Lee C Kang GH Lee HS. Evaluation of an eight marker-panel including long mononucleotide repeat markers to detect microsatellite instability in colorectal, gastric, and endometrial cancers. BMC Cancer. (2023) 23:1100. doi: 10.1186/s12885-023-11607-6, PMID: 37953261 PMC10641958

[18] Malapelle U Parente P Pepe F De Luca C Pisapia P Sgariglia R. Evaluation of micro satellite instability and mismatch repair status in different solid tumors: A multicenter analysis in a real world setting. Cells. (2021) 10:1878. doi: 10.3390/cells10081878, PMID: 34440647 PMC8391221

[19] Dedeurwaerdere F Claes KB Van Dorpe J Rottiers I van der Meulen J Breyne J. Comparison of microsatellite instability detection by immunohistochemistry and molecular techniques in colorectal and endometrial cancer. Sci Rep. (2021) 11:12880. doi: 10.1038/s41598-021-91974-x, PMID: 34145315 PMC8213758

[20] Bartley AN Mills AM Konnick E Overman M Ventura CB Souter L. Mismatch repair and microsatellite instability testing for immune checkpoint inhibitor therapy: guideline from the college of american pathologists in collaboration with the association for molecular pathology and fight colorectal cancer. Arch Pathol Lab Med. (2022) 146:1194–210. doi: 10.5858/arpa.2021-0632-CP, PMID: 35920830

[21] Stelloo E Jansen AML Osse EM Nout RA Creutzberg CL Ruano D. Practical guidance for mismatch repair-deficiency testing in endometrial cancer. Ann Oncol. (2017) 28:96–102. doi: 10.1093/annonc/mdw542, PMID: 27742654

[22] Gargett CE Nguyen HPT Ye L. Endometrial regeneration and endometrial stem/progenitor cells. Rev Endocr Metab Disord. (2012) 13:235–51. doi: 10.1007/s11154-012-9221-9, PMID: 22847235

[23] Helderman NC Haupt S Strobel F Bohaumilitzky L Van Wezel T Morreau H. Microsatellite peak shifts in polymerase chain reaction-based fragment length data correlate with microsatellite instability degree and vary with mismatch repair gene defects and tumor size. JCO Precis Oncol. (2025) 9:e2400731. doi: 10.1200/PO-24-00731, PMID: 40526878

[24] Verma L Kane M Brassett C Schmeits J Evans D Kolodner R. Mononucleotide microsatellite instability and germline MSH6 mutation analysis in early onset colorectal cancer. J Med Genet. (1999) 36:678–82. doi: 10.1136/jmg.36.9.678, PMID: 10507723 PMC1734424

[25] Takehara Y Nagasaka T Nyuya A Haruma T Haraga J Mori Y. Accuracy of four mononucleotide-repeat markers for the identification of DNA mismatch-repair deficiency in solid tumors. J Transl Med. (2018) 16:5. doi: 10.1186/s12967-017-1376-4, PMID: 29329588 PMC5767035

[26] Graham RP Kerr SE Butz ML Thibodeau SN Halling KC Smyrk TC. Heterogenous MSH6 loss is a result of microsatellite instability within MSH6 and occurs in sporadic and hereditary colorectal and endometrial carcinomas. Am J Surg Pathol. (2015) 39:1370–6. doi: 10.1097/PAS.0000000000000459, PMID: 26099011

[27] Pan S Cox H Willmott J Mundt E Gorringe H Landon M. Discordance between germline genetic findings and abnormal tumor immunohistochemistry staining of mismatch repair proteins in individuals with suspected Lynch syndrome. Front Oncol. (2023) 13:1069467. doi: 10.3389/fonc.2023.1069467, PMID: 36793599 PMC9923021

[28] Dudley B Brand RE Thull D Bahary N Nikiforova MN Pai RK. Germline MLH1 mutations are frequently identified in lynch syndrome patients with colorectal and endometrial carcinoma demonstrating isolated loss of PMS2 immunohistochemical expression. Am J Surg Pathol. (2015) 39:1114–20. doi: 10.1097/PAS.0000000000000425, PMID: 25871621

[29] Jiang W Cai M-Y Li S-Y Bei J-X Wang F Hampel H. Universal screening for Lynch syndrome in a large consecutive cohort of Chinese colorectal cancer patients: High prevalence and unique molecular features. Int J Cancer. (2019) 144:2161–8. doi: 10.1002/ijc.32044, PMID: 30521064

