# Supplementary material for: Evaluation of microsatellite instability patterns in mismatch repair deficiency: a retrospective analysis of 285 endometrial cancers
Source: Front Immunol. 2025 Sep 17;16:1628979. doi: 10.3389/fimmu.2025.1628979 (PMC12484153; doi:10.3389/fimmu.2025.1628979)
Supplement: Supplementary file 1 [file Table1.docx]

Supplementary table 1. Frequency of minimal microsatellite shifts at single nucleotide and dinucleotide loci (n=285).

|  |  | Total |
| --- | --- | --- |
|  |  | n=285 |
| MSI loci |  |  |
| single nucleotide | BAT26 | 143 (50.1%) |
|  | BAT25 | 167 (58.5%) |
| dinucleotide | D5S346 | 35 (12.2%) |
|  | D17S250 | 24 (8.4%) |
|  | D2S123 | 20 (7%) |

MSI, microsatellite instability.

Supplementary table 2. Frequency of minimal microsatellite shifts in MMR protein deficient patients (n=231).

|  | Total |
| --- | --- |
|  | n=231 |
| MMR protein deficient |  |
| MLH1 and PMS2 | 145 (62.8%) |
| MSH2 and MSH6 | 36 (15.6%) |
| MSH6 | 39 (16.9%) |
| PMS2 | 11 (4.7%) |

MMR, mismatch repair.
